# A Peptide-Centric DIA-NN Reanalysis Uncovers Structurally Coherent Salivary Signatures of Type 2 Diabetes

**DOI:** 10.3390/ijms27042040

**Published:** 2026-02-22

**Authors:** Rui Vitorino

**Affiliations:** 1 iBiMED, Department of Medical Sciences, University of Aveiro, 3810-193 Aveiro, Portugal; rvitorino@ua.pt; 2 RISE, Department of Surgery and Physiology, Faculty of Medicine, University of Porto, 4099-002 Porto, Portugal

**Keywords:** peptide-centric proteomics, bottom-up proteomics, structure-informed peptide analysis

## Abstract

Type 2 diabetes (T2D) causes systemic metabolic and inflammatory changes that affect the oral cavity, but salivary molecular markers remain poorly characterized. A peptide-centric reanalysis of salivary proteomics data was performed using DIA-NN for peptide-level quantification, without collapsing peptide signals into protein-level summaries. Although the qualitative peptide repertoire was largely conserved between T2D and control samples (>96% overlap), T2D showed coordinated quantitative changes in specific peptide subsets. Differentially abundant peptides primarily originated from complement C3, alpha-2-macroglobulin, serotransferrin, mucins, apolipoproteins, and hemoglobin, with a significant enrichment of oxidized cysteine-containing peptides, indicating redox imbalance and low-grade inflammation. Structural analysis with AlphaFold showed that T2D-associated peptides are located in solvent-exposed and conformationally dynamic regions of proteins. These findings suggest that disease specificity in diabetic saliva occurs mainly at the peptide level, offering mechanistic insight into non-invasive biomarker identification and longitudinal disease monitoring.

## 1. Introduction

Type 2 diabetes (T2D) is a systemic metabolic condition characterized by persistent hyperglycemia, mild inflammation, and progressive vascular and epithelial impairment. While blood-based biomarkers remain the clinical standard for assessing metabolic health, there is growing interest in saliva as a non-invasive diagnostic medium that can reflect both local and systemic biochemical changes [1]. Saliva captures the contributions of salivary glands, gingival crevicular fluid, mucosal surfaces, and microvasculature, serving as a sensitive indicator of inflammatory status, epithelial integrity, and metabolic stress. Beyond diagnostics, the molecular composition of saliva is increasingly recognized as relevant to oral mucosal barrier function and to the design of mucosa-mimetic biomaterials, where hydration, lubrication, immunological compatibility, and redox balance are critical parameters. Consequently, salivary proteomics has emerged as a valuable adjunct for monitoring metabolic diseases, particularly in contexts requiring frequent sampling or patient-friendly collection methods [2,3].

Recent proteomic and metaproteomic studies have shown that the salivary molecular profile of individuals with type 2 diabetes is altered at multiple levels, including changes in immune-related proteins, mucins, and microbial components. Although these protein-level studies have provided important insights, they primarily reflect aggregate abundance changes and may obscure more subtle molecular variations present at the peptide level [3,4]. The conceptual and methodological basis for peptide-centric analysis has been clearly outlined in the proteomics literature. Previous work showed that a peptide-focused reanalysis of bottom-up proteomics datasets can reveal biologically and clinically significant features often hidden in traditional protein-centric approaches due to protein inference, peptide averaging, and statistical attenuation [5]. Through a reanalysis of proteomics data from prostate cancer, the authors showed that a substantial proportion of statistically significant peptides correspond to proteins that do not reach significance at the protein level, thereby uncovering disease-associated molecular signatures that would otherwise remain undetected. This framework explicitly defines digestion-derived peptides as analytical features rather than native endogenous entities, establishing peptide-centric analysis as a complementary and structurally informative dimension within bottom-up proteomics, distinct from traditional endogenous peptidomics [5,6]. Peptide-focused methods, especially when combined with modern data-independent acquisition (DIA) techniques and neural network-assisted analysis, enable the precise and consistent quantification of peptide fragments that indicate proteolytic activity, epithelial integrity, redox state, and vascular permeability. These peptide-level features may serve as early molecular markers of tissue remodeling in metabolic disorders; however, they remain largely unexplored in diabetic saliva [5,6]. The mechanical and biochemical properties of bioinks, such as hydration, lubrication, diffusivity, immunological compatibility, and redox stability, are significantly affected by the peptides and proteins present in the oral environment [7]. Structural modeling techniques, such as AlphaFold, now allow for the correlation of peptide abundance changes with predicted physicochemical patterns, providing greater insight into how disease-related secretions may interact with engineered tissues [8,9,10].

This paper defines peptide-centric analysis as the quantitative and sequence-level interpretation of tryptic peptides produced by bottom-up proteomics. The objective is not to attribute independent biological regulation or inherent structural function to individual peptides, but to use peptide-level resolution to reveal intra-protein heterogeneity and physicochemical patterns that are often attenuated or lost in protein-centric analyses. While Samodova et al. [11] provided a comprehensive protein-level and metaproteomic characterization of diabetic saliva, the present peptide-centric reanalysis of the same dataset enables the direct assessment of molecular features that are not accessible through protein-level aggregation alone. The researchers conducted a comprehensive protein-level and taxonomic characterization of the host and microbial components in saliva from individuals with T2D, creating a significant reference resource for T2D-related molecular and microbiome changes. The present study deliberately uses a more focused yet distinct analytical approach, restricting the analysis to human-derived peptides and examining disease-related changes at the peptide level rather than at the full protein or microbial taxonomic level. Through a peptide-focused re-evaluation of the same raw DIA-PASEF data and integration of AlphaFold-derived structural modeling, it aims to obtain mechanistic and physicochemical insights that are fundamentally unattainable through protein-level or taxonomic assessments. This strategy does not seek to reinvent the previously characterized salivary proteome landscape, but to expand it by clarifying how diabetes-related proteolytic patterns and peptide architectures may influence mucosal barrier function and the development of mucosa-mimetic biomaterials. The two studies address complementary biological questions using the same high-quality data, together providing a multifaceted understanding of diabetic saliva. All the experimental data analyzed in this study were previously generated and published by Samodova et al. [11], and the present work constitutes a secondary computational reanalysis of the publicly available DIA-PASEF dataset (PXD051453) using a distinct peptide-centric and structure-oriented analytical framework. The aim of this study was to reanalyze publicly available salivary DIA-PASEF proteomics data from individuals with type 2 diabetes using a peptide-centric DIA-NN workflow and structure-informed mapping to determine whether peptide-level quantification reveals disease-associated molecular patterns that are obscured by protein-level aggregation. It sought to identify coordinated peptide signatures, define their physicochemical and structural locations within parent proteins, and evaluate their significance in relation to chronic metabolic stress in the oral environment.

## 2. Results

### 2.1. Peptide Overlap and Conserved Salivary Backbone in T2D

**Figure 1:**
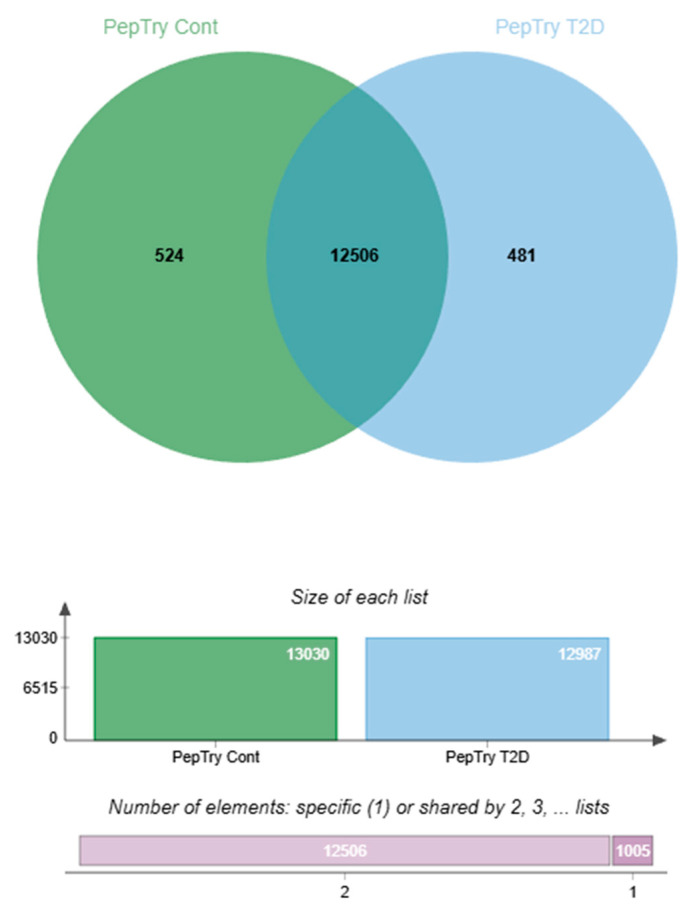
Peptide-level overlap (DIA-NN reanalysis) Venn diagram showing the intersection of peptides identified by trypsin-specific DIA-NN analysis in the control (CTRL) and T2D saliva samples. A total of 12,506 peptides were shared by both groups, representing the conserved core salivary peptidome. Additionally, 524 peptides were found only in the CTRL samples, and 481 peptides were unique to the T2D group. In total, 13,030 peptides were identified in the CTRL samples and 12,987 in the T2D samples. The large proportion of shared peptides indicates a stable salivary peptide repertoire, with disease-related differences mainly reflected by quantitative changes within this common peptide pool rather than by qualitative presence-absence differences. Generated from peptide-centric DIA-NN reanalysis of raw data (PXD051453); figures are not reproduced from Samodova et al. [11].

Peptide-centric DIA-NN profiling showed a largely conserved salivary peptide repertoire between normoglycemic controls (CTRL) and individuals with T2D. As shown in Figure 1, a total of 12,506 peptides were shared between the CTRL and T2D samples, representing 96.0% of peptides identified in the CTRL samples and 96.3% of those identified in the T2D samples. Only a small percentage of peptides was unique to each group, with 524 peptides found only in CTRL (4.0%) and 481 peptides found only in T2D (3.7%), out of 13,030 peptides in CTRL and 12,987 in T2D (Figure 1).

This significant overlap suggests that T2D does not substantially alter the qualitative structure of the salivary peptidome but instead causes quantitative changes within a largely stable peptide framework. Notably, the peptides that most effectively distinguished the groups in subsequent multivariate analyses were primarily drawn from the shared peptide pool, indicating that disease-associated signatures are driven by differences in the abundance of biologically relevant peptides rather than the appearance of disease-specific peptide sequences.

Functional enrichment analysis of peptides associated with CTRL samples revealed a profile mainly characterized by epithelial structure and homeostasis, including keratinization, keratinocyte development, epithelial cell differentiation, cytoskeleton organization, and supramolecular fiber organization. A complete list of significantly enriched biological processes for CTRL- and T2D-associated peptide clusters is provided in Supplementary Table S1. These mechanisms reflect the ongoing regeneration and barrier-preserving functions of the oral epithelium in healthy conditions. The increased enrichment of pathways related to protein folding, translation, organelle organization, intracellular transport, and vesicle-mediated secretion further supports the integrity of proteostasis and secretory function in CTRL saliva.

In contrast, peptides associated with the T2D cohort showed enrichment in biological processes related to oxidative stress response, detoxification, and tissue repair, including hydrogen peroxide catabolism, reactive oxygen species (ROS) metabolism, cellular oxidant detoxification, response to toxic agents, hemostasis, wound healing, and vesicle-mediated transport. This functional shift is consistent with the chronic low-grade inflammation, redox imbalance, and impaired tissue repair characteristic of type 2 diabetes pathogenesis. These findings collectively indicate that T2D-related salivary changes occur within a preserved epithelial framework, signifying functional reprogramming and stress adaptation rather than a loss of epithelial identity.

### 2.2. Differential Peptide Abundance and Multivariate Structure of the T2D Salivary Peptidome

**Figure 2:**
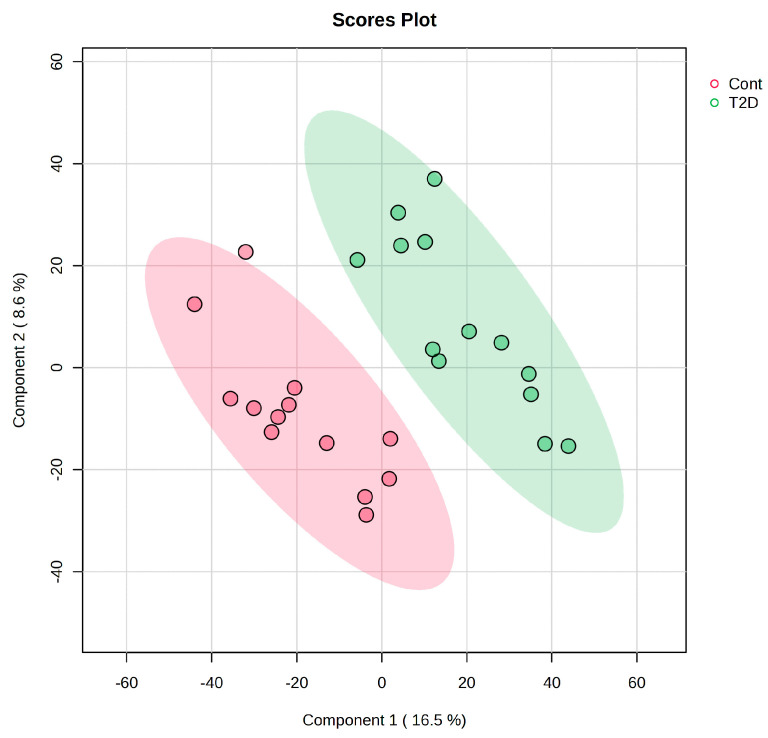
PLS score plot illustrating peptide-centric DIA-NN quantification in saliva samples. PLS with trypsin-specific, peptide-level DIA-NN quantification shows the distribution of control (CTRL; red) and type 2 diabetes (T2D; green) saliva samples. The distinction between groups is most evident in Component 1, which explains 16.5% of the variation, while Component 2 accounts for 8.6% of the variance, based on peptide-centric DIA-NN quantification. Shaded ellipses indicate the 95% confidence intervals for each group. Samples 452, 453, 472, and 473, identified as multivariate outliers in an initial PLS, were excluded from this study to improve model’s stability and interpretability. Generated from peptide-centric DIA-NN reanalysis of raw data (PXD051453); figures are not reproduced from Samodova et al. [11].

Peptide-centric quantification from trypsin-specific DIA-NN analysis underwent unsupervised multivariate analysis to assess global structure and disease-related variation in the salivary peptidome. Preliminary principal component analysis (PCA) showed clear differentiation between the CTRL and T2D samples along the first principal component (PC1), which explained 16.5% of the total variance, while the second component (PC2) accounted for an additional 8.6% (Figure 2). Four samples (IDs 452, 453, 472, and 473) consistently deviated from their respective group clusters and were classified as multivariate outliers. Because of their disproportionate impact on the PCA space, these data were excluded from further multivariate analyses to improve model robustness and interpretability. After outlier removal, the remaining samples showed greater intra-group cohesiveness and a more distinct separation between the CTRL and T2D groups, supporting the presence of a disease-associated peptide signature at the peptide level.

**Figure 3:**
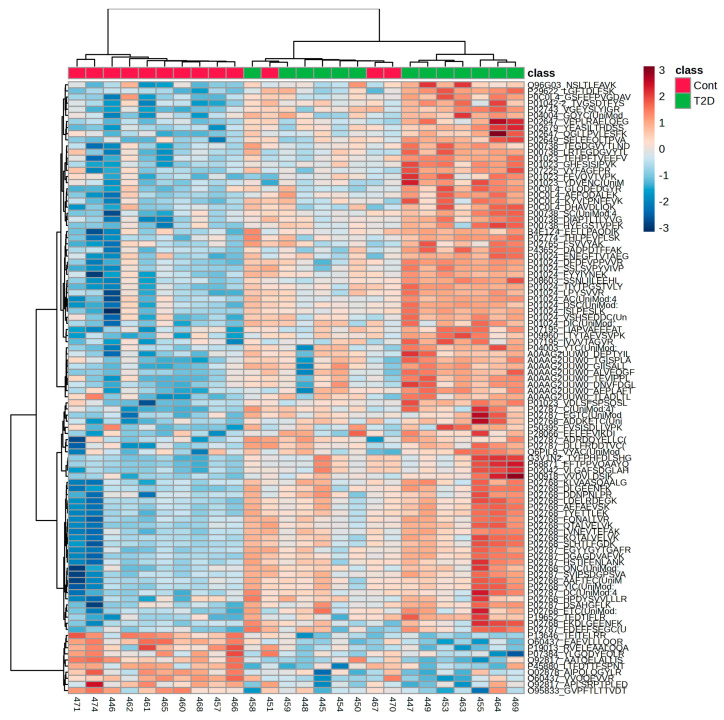
Hierarchical clustering heatmap of peptide-centric DIA-NN profiles in saliva. The heatmap shows the unsupervised hierarchical clustering of peptide-level abundances from trypsin-specific DIA-NN analysis in control (CTRL; red) and type 2 diabetes (T2D; green) saliva samples. Columns represent individual samples. Rows represent tryptic peptides (not proteins). Peptide abundances were log_2_-transformed and Z-score normalized across samples before clustering. Dendrograms display the hierarchical clustering of peptides and samples using Euclidean distance and complete linkage. The color gradient indicates relative peptide abundance (blue indicates a lower quantity; red indicates a higher abundance). The upper annotation bar shows the sample class. The heatmap reveals coordinated peptide abundance patterns that distinguish CTRL and T2D samples, reflecting disease-related quantitative changes in the salivary peptidome. Generated from the peptide-centric DIA-NN reanalysis of raw data (PXD051453); figures are not reproduced from Samodova et al. [11].

The unsupervised hierarchical clustering of peptide abundances further supported these findings, revealing distinct and coherent peptide clusters that separated the T2D from CTRL samples. A significant cluster of peptides showed persistently elevated relative abundance in the saliva of individuals with T2D, forming a key disease-associated module. These peptides were primarily derived from proteins involved in innate immunity, oxidative stress modulation, protease–antiprotease balance, lipid transport, and vascular permeability. This includes peptides from P01023 (alpha-2-macroglobulin), P01024 (complement C3), P02768 (albumin), P02787 (serotransferrin), P00738 (haptoglobin), and P0C0L4, as well as a group of peptides corresponding to A0AAG2UUW0 (Figure 3).

Several T2D-enriched peptides notably contained cysteine residues with oxidation-related UniMod annotations, consistent with the oxidative stress-dominated biological processes identified in functional enrichment analysis. The repeated detection of multiple peptides from the same parent proteins, all showing consistent abundance trends, suggests a physiologically driven signal rather than random peptide-level variation. The cysteine modifications found in T2D-enriched peptides mostly matched UniMod:35 (+15.9949 Da), as expected from reversible cysteine oxidation observed during DIA-NN searching. Carbamidomethylation (fixed) resulted from the chemical used to prepare the samples. No systematic increase in higher-order irreversible cysteine oxidations, such as sulfinic or sulfonic acid forms, was detected. Aside from methionine oxidation (UniMod:35) and N-terminal acetylation, no other post-translational modifications showed consistent differences between the CTRL and T2D groups. This indicates that disease-related changes are primarily due to differences in peptide abundance rather than in the types of PTMs. In contrast, a smaller and more compact cluster of peptides had a higher relative abundance in the CTRL samples. The CTRL-associated peptides, P13646_TEITELRR, O60437_EAEVLLLQQR, P19013_RVELEAALQQA, P07384_YLGQDYEQLR, Q92817_AATQELALLIS, P45880_LTFDTTFSPNT, Q02878_AIPQLQGYLR, O60437_VVQQEVVR, Q92817_APLSRPTPLED, and O95833_GVPFTLTTVDT, are mainly linked to core cellular metabolism, structural stability, and epithelial maintenance, consistent with the CTRL-specific enhancement of keratinization and epithelial differentiation pathways.

The combined multivariate and clustering analyses indicate that salivary changes associated with T2D result from the coordinated quantitative regulation of specific peptide subsets, rather than isolated peptide anomalies or a broad disruption of salivary composition. These findings highlight that peptide-centric analysis uncovers disease-relevant molecular changes that are partially masked in protein-level assessments, providing a more nuanced and mechanistically insightful view of salivary alterations in type 2 diabetes. To directly compare peptide-centric and protein-centric interpretations, representative proteins identified in both this reanalysis and the original work by Samodova et al. [11] were examined. For complement C3, several peptides showed coordinated enrichment in T2D saliva, with median log_2_ fold changes between approximately 0.6 and 0.9 (*p* < 0.01 at the peptide level); however, the corresponding protein-level abundance reported by Samodova et al. [11] did not reach statistical significance. Alpha-2-macroglobulin and transferrin also displayed consistent increases at the peptide level for multiple peptides (log_2_ fold change ~0.4–0.8), although these signals were diminished by protein-level aggregation. These examples illustrate how peptide averaging in protein-centric analyses can obscure disease effects that are specific to certain regions but may be detected using peptide-centric DIA-NN analysis.

### 2.3. Structure-Informed Physicochemical Analysis of Differentially Abundant Peptide Sequences

Some of the proteins examined here, such as complement C3, alpha-2-macroglobulin, and transferrin, have partial structures that have been experimentally determined and are stored in the Protein Data Bank. However, these structures typically represent isolated domains, activated segments, or complex-specific conformations, and lack continuous full-length models suitable for systematic peptide mapping. To obtain full-length structural predictions of proteins with residue-level confidence metrics (pLDDT and PAE), AlphaFold was used. This enabled the consistent placement of all differentially abundant peptides within a single structural framework. AlphaFold modeling was used as a sequence-informed physicochemical interpretation tool to enhance peptide-centric quantitative analysis, rather than as a predictor of the stable native folding of isolated tryptic peptides. Because digestion-derived peptides are short, structural predictions were analyzed for conformational propensity, flexibility, amphipathicity, charge distribution, and solvent accessibility, providing context for how disease-associated peptide fragments may reflect modified protein states and microenvironmental conditions in saliva.

Structural analysis of complement C3-derived peptides (P01024):

Complement C3 (P01024; 1663 amino acids) is a key integrator of innate immunity, inflammation, and metabolic stress. AlphaFold’s prediction of full-length C3 shows a highly modular structure, consisting of multiple macroglobulin (MG) domains, CUB domains, and a C-terminal C345C domain connected by flexible regions. The predicted aligned error (PAE) map shows high confidence within individual domains and lower confidence in inter-domain regions, consistent with inherent conformational flexibility. These cysteine modifications correspond mainly to UniMod:35 oxidative events rather than irreversible overoxidation.

Mapping T2D-enriched peptides onto the predicted structure shows that the distinguishing C3-derived peptides (e.g., ENEGFTVTAEG, DFDFVPPVVR, SSLSVPYVIVP, TIYTPGSTVLY, LPYSVVR, and various cysteine-containing UniMod-modified sequences) are mainly located in surface-exposed and conformationally dynamic regions, rather than within the buried structural core. This distribution increases their analytical detectability and suggests that quantitative differences reflect modified protein exposure, activation status, or local processing, rather than random degradation. It is important to note that tryptic digestion was performed under fully denaturing conditions, so the efficacy of proteolytic cleavage was not limited by the accessibility of the native structure. Therefore, the preferential localization of T2D-enriched peptides to solvent-exposed and flexible regions is not considered indicative of differential digestion but rather reflects upstream biological processes that affect the protein abundance, activation state, redox modification, or tissue leakage before sample processing. Structural mapping is used solely as an interpretative framework to contextualize peptide-level quantitative changes, indicating that disease-associated signals mainly originate from protein regions that are biologically active and chemically sensitive in vivo.

**Figure 4:**
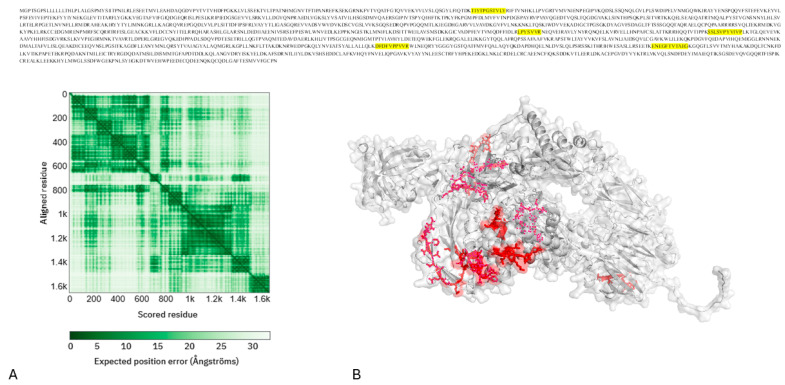
Structural context of complement C3 (P01024) and mapping of peptides in type 2 diabetes. A light gray ribbon with a semi-transparent surface overlay shows the full-length structure of human Complement C3 as predicted by AlphaFold. Tryptic peptides more common in T2D (such as ENEGFTVTAEG, DFDFVPPVVR, and TIYTPGSTVLY) are shown in yellow and stick form. This image, together with the clear surface, shows that these peptides are located on surface loops that are accessible to solvents and undergo conformational changes. The projected aligned error (PAE) (**A**) heatmap indicates the confidence in each domain and the flexibility of the interdomain regions (**B**).

Many peptides contain oxidized cysteine residues that, when analyzed using the AlphaFold model, correspond to solvent-accessible regions near domains involved in complement activation and regulatory interactions. This finding is consistent with a redox-modulated complement environment, a recognized feature of persistent low-grade inflammation and oxidative stress in type 2 diabetes. The clustering of discriminatory peptides in flexible and exposed regions suggests that the salivary C3 peptide signature reflects the functional remodeling of complement activity, rather than uniform changes in the overall protein abundance (Figure 4).

Peptides of alpha-2-macroglobulin (A2M; P01023) and the balance between proteases and antiproteases:

Two peptides from alpha-2-macroglobulin (A2M; P01023)—IAQWQSFQLEGGLK and LVHVEEPHTETVRK—were consistently elevated in the saliva from individuals with T2D. A2M is a major plasma protease inhibitor that regulates proteolytic activity during inflammation, tissue remodeling, and vascular permeability. The AlphaFold modeling of full-length A2M (1474 amino acids) shows a multi-domain, conformationally flexible structure, with high confidence in individual domains and lower confidence in the hinge-like regions associated with its protease-trapping mechanism.

**Figure 5:**
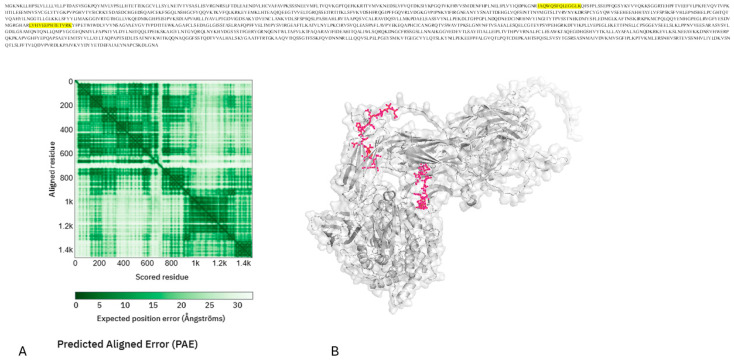
Mapping of T2D-related peptides in alpha-2-macroglobulin (P01023). AlphaFold predicted (**A**) the structure of alpha-2-macroglobulin, shown as a light gray ribbon (**B**). The yellow stick illustration shows the side-chain orientation of the T2D-enriched peptides, including IAQWQSFQLEGGLK and LVHVEEPHTETVRK. The addition of a semi-transparent surface provides direct visual evidence that these peptides map to regions of the A2M protein that are structured and solvent exposed. This demonstrates that changes in salivary protein abundance in people with T2D are linked to protein regions that are chemically active and accessible in the oral cavity.

Both peptides map to structured, solvent-exposed regions of the A2M structure, rather than to disordered terminal segments. Their coordinated enrichment indicates a physiologically consistent signal representing a modified protease–antiprotease balance and increased inflammatory turnover in T2D, rather than random peptide variation. This interpretation aligns with functional enrichment findings that highlight pathways related to wound healing, hemostasis, and stress response, supporting a model of protease-mediated inflammatory remodeling in the diabetic salivary environment (Figure 5).

AlphaFold predicted the structure of alpha-2-macroglobulin, shown as a light gray ribbon. The red stick illustration shows the side-chain orientation of the T2D-enriched peptides, including IAQWQSFQLEGGLK and LVHVEEPHTETVRK. The addition of a semi-transparent surface provides direct visual evidence that these peptides map to regions of the A2M protein that are structured and solvent exposed. This demonstrates that changes in salivary protein abundance in people with T2D are linked to protein regions that are chemically active and accessible in the oral cavity.

**Figure 6:**
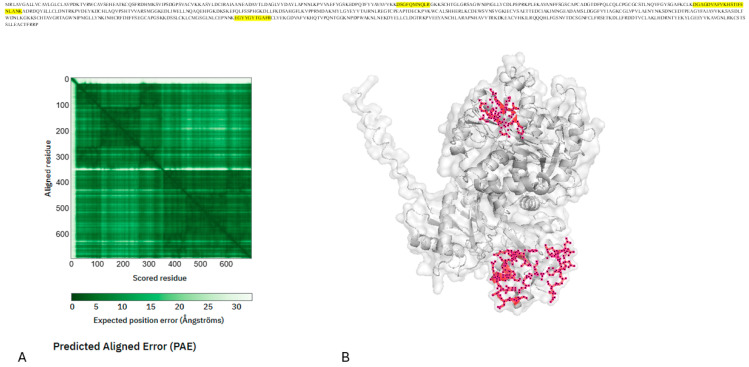
Peptide cluster linked to serotransferrin (P02787). The AlphaFold prediction of transferrin (698 amino acids) shows a well-structured bilobal conformation with high overall confidence (**A**). The frequent detection of oxidized cysteine-containing peptides is chemically plausible, as these residues are expected to be solvent-accessible and located in regions susceptible to oxidative modification (**B**). Transferrin is central to iron transport and redox balance, and its peptide-level enrichment, along with frequent cysteine oxidation, strongly suggests the disruption of the iron–oxidative stress axis in the saliva of individuals with T2D.

Peptide cluster of serotransferrin (P02787) and dysregulation of redox iron:

Hierarchical clustering identified a significant T2D-associated peptide module predominantly composed of serotransferrin (TF; P02787). Multiple transferrin-derived peptides showed consistent elevation in T2D saliva, including both unmodified sequences (e.g., DGAGDVAFVK, DSGFQMNQLR, EGYYGYTGAFR, and HSTIFENLANK) and a broad range of cysteine-containing peptides annotated with UniMod oxidation (Figure 6).

The repeated detection of multiple peptides from the same parent protein, all showing consistent abundance patterns, indicates a protein-level biological signal that is more sensitively detected at the peptide level. This finding closely corresponds with the enhancement of hydrogen peroxide metabolism, detoxification, and oxidative stress response pathways observed in the T2D cohort.

The AlphaFold-predicted ribbon structure of serotransferrin shows T2D-associated tryptic peptides as red sticks. These peptides include DGAGDVAFVK, DSGFQMNQLR, EGYYGYTGAFR, and HSTIFENLANK. The semi-transparent surface overlay shows that these enriched peptides are generally located on both the N- and C-lobes in regions exposed to solvent. This structural placement supports the conclusion that these modules are highly susceptible to the metabolic changes and abundance fluctuations observed in the T2D cohort.

Integrative structural motifs in T2D-related peptides:

Peptides with differential abundance among proteins were classified into a limited set of recurring physicochemical categories: (i) flexible, loop-rich segments from mucins and barrier-associated proteins; (ii) amphipathic helices characteristic of lipid-binding and transport proteins; (iii) compact interaction motifs from complement and protease inhibitors; and (iv) helix-rich globin segments containing redox-active residues. These persistent patterns indicate that peptide-level changes in T2D saliva are systematic, reflecting a coordinated reconfiguration of mucosal lubrication, immune regulation, vascular permeability, and oxidative balance. Importantly, AlphaFold modeling is used not to infer the independent biological function of peptides, but to contextualize the relationship between the structural and physicochemical properties of peptide sequences and disease-related changes in protein accessibility and processing. Overall, structure-informed peptide analysis establishes a mechanistic link between quantitative peptidomics and functional biology, highlighting that type 2 diabetes alters the salivary microenvironment through specific, structurally consistent molecular modifications rather than broad proteome disruption.

## 3. Discussion

This study provides a nuanced perspective on how T2D alters the oral molecular environment by examining digestion-derived peptides as high-resolution analytical indicators of protein abundance, accessibility, and tissue leakage, rather than relying solely on intact protein-level summaries. All the analyzed peptides originate from controlled Lys-C/trypsin digestion and thus reflect the analytical characteristics of bottom-up proteomics, not endogenous bioactive peptides. Therefore, variations at the peptide level are not interpreted as evidence of peptide-specific biological regulation or changes in in vivo proteolysis. Peptide-centric quantification serves as a sensitive analytical approach to detect intra-protein heterogeneity and physicochemical gradients that indicate disease-related changes in protein exposure, redox status, immune activation, and vascular permeability. A key finding is the significant conservation of the qualitative salivary peptide repertoire between individuals with T2D and those who are normoglycemic. Over 96% of identified peptides were shared across groups, indicating that diabetes does not fundamentally alter the peptide sequences present in saliva. Disease-related information primarily arises from quantitative changes within a consistent peptidomic framework. This finding is biologically consistent with the chronic and gradually progressing nature of T2D, in which functional reprogramming and metabolic stress occur before significant tissue deterioration. It explains the modest or inconsistent discrimination observed in many previous protein-centric salivary studies: disease specificity mainly results from coordinated abundance changes in particular peptide subsets, rather than the appearance or disappearance of proteins or peptides.

The changes in the peptide levels detected in the saliva of individuals with T2D are asymmetric and selective, not universal. While numerous differentially abundant peptides originate from plasma-associated proteins typically linked to inflammation and vascular leakage (e.g., complement C3, alpha-2-macroglobulin, transferrin, hemoglobin, and apolipoproteins), several features suggest that a purely nonspecific inflammatory interpretation is insufficient. First, more than 96% of the qualitative peptide repertoire is preserved between CTRL and T2D, indicating that diabetes does not cause widespread proteome turnover but rather selective quantitative remodeling. Second, enrichment is confined to specific peptide subsets within these proteins, rather than showing uniform increases across all regions. This demonstrates intra-protein heterogeneity that cannot be explained by passive plasma leakage alone. Third, enriched peptides tend to map to regions that are redox-sensitive, flexible, and solvent-exposed. This suggests that chronic metabolic stress induces molecular remodeling in a specific manner. Collectively, these characteristics support a scenario in which T2D enhances low-grade inflammatory pathways while imposing disease-specific structural and physicochemical signatures on salivary proteins, rather than merely reflecting generic inflammation. A limited set of peptides shows significant enrichment, while fewer peptides are reduced. This pattern does not support indiscriminate proteolysis or the broad dysregulation of salivary secretion. Instead, it indicates targeted molecular restructuring associated with epithelial turnover, immune activation, oxidative stress, and microvascular permeability. Enriched peptides primarily originate from proteins involved in mucosal barrier function (mucins), lipid transport (apolipoproteins), innate immunity and protease regulation (complement components and alpha-2-macroglobulin), iron and redox homeostasis (serotransferrin), and blood-derived proteins (hemoglobin) [12]. The consistent response of several peptides from the same parent proteins strongly suggests a physiologically directed signal rather than random peptide-level variation [13].

The peptide-centric framework significantly improves the quantitation of proteins previously identified in diabetic saliva. It reveals that these proteins are remodeled in specific ways not apparent in protein-level summaries. Complement C3, alpha-2-macroglobulin, and transferrin do not exhibit uniform changes in abundance; rather, only certain peptide regions, typically those that are flexible, solvent exposed, or redox sensitive, are consistently enriched. This variation within the protein indicates specific structural and physicochemical changes associated with inflammation, vascular permeability, and oxidative stress, rather than simple increases in the total protein concentration. These findings shift our understanding of the changes in diabetic saliva from bulk protein leakage to region-specific molecular remodeling, providing better insight into how systemic metabolic stress manifests on mucosal surfaces. Unlike protein-level analysis, peptide-centric quantification reveals intra-protein heterogeneity that is typically hidden [14]. Proteins such as complement C3, alpha-2-macroglobulin, and transferrin do not act as uniform entities; instead, specific peptide regions show significant differences in abundance. These regions often correspond to flexible, solvent-accessible, or physiologically relevant segments, explaining why biologically important disease signatures may not reach significance at the protein level, even when they are robust and reproducible at the peptide level [15]. Peptide-centric analysis should be viewed not as a replacement for protein-centric proteomics, but as an enhanced analytical dimension that reveals disease-relevant molecular gradients within proteins. In practical terms, several proteins that were not significantly altered at the protein level in the original analysis (e.g., complement C3, alpha-2-macroglobulin, and transferrin) showed multiple peptides with statistically significant abundance changes and moderate effect sizes, demonstrating that protein-level aggregation suppresses localized disease signals.

A distinctive feature of the T2D-associated peptide signature is the prevalence of cysteine-containing peptides with oxidation-related UniMod annotations. These peptides are consistently elevated in the saliva of individuals with T2D and closely align with functional enrichment findings related to hydrogen peroxide metabolism, reactive oxygen species detoxification, wound healing, and stress response pathways. This pattern does not indicate the selective regulation of certain peptides or unregulated proteolysis. Instead, it reflects a redox-altered salivary microenvironment where oxidative changes predominantly affect solvent-exposed and conformationally dynamic regions of proteins. This finding is fully consistent with the chronic low-grade inflammation and oxidative stress that characterize the pathogenesis of T2D [16,17].

AlphaFold structural modeling provides a crucial interpretive dimension by placing peptide-level changes within the three-dimensional context of their parent proteins [18]. In this study, AlphaFold is not used to infer the independent folding or biological function of individual peptides. Instead, structural predictions clarify the physical properties of peptide sequences, such as flexibility, solvent accessibility, amphipathicity, and their susceptibility to chemical modification. When T2D-enriched peptides are mapped onto whole protein models, they consistently localize to surface-exposed and conformationally dynamic regions, increasing their analytical detectability and vulnerability to disease-related redox and inflammatory processes [19]. In this work, the phrase “structurally coherent salivary signatures” refers to the observation that peptides with varying abundance levels do not distribute randomly along parent proteins but instead cluster within specific structural contexts. T2D-enriched peptides preferentially localize to solvent-accessible and conformationally dynamic regions and consistently display common physicochemical features, such as flexible loop segments, amphipathic helices, and redox-sensitive cysteine-containing motifs. Quantitatively, this coherence is supported by (i) the consistent direction of abundance changes across multiple peptides derived from the same protein, (ii) the enrichment of peptides in regions with lower AlphaFold pLDDT confidence or higher PAE, indicating structural flexibility, and (iii) the convergence of peptides into a limited number of recurring structural categories rather than being evenly distributed across all protein sequences. Together, these features define “structural coherence” as synchronized peptide-level changes within specific protein regions, rather than as isolated or random peptide modifications.

Different proteins yield differentially abundant peptides that cluster into a limited set of recurring structural motifs: (i) flexible, loop-rich segments from mucins and barrier-associated proteins that promote hydration and lubrication; (ii) amphipathic α-helical regions characteristic of lipid-binding and transport proteins; (iii) compact interaction motifs from complement components and protease inhibitors involved in immune regulation; and (iv) helix-rich segments from globins that retain redox-active residues [16,20,21]. These recurring patterns indicate that peptide-level changes in T2D saliva are systematic, reflecting a coordinated reorganization of molecular features that collectively influence mucosal lubrication, immune surveillance, vascular permeability, and oxidative balance.

From a translational perspective, peptide-focused salivary analysis offers distinct advantages. Many discriminating peptides show abundance changes that exceed the dynamic range typically observed at the protein level, suggesting greater sensitivity for detecting early or subclinical metabolic stress. Because saliva collection is non-invasive and suitable for repeated sampling, peptide-level signatures may be especially valuable for the longitudinal monitoring of disease progression or therapeutic response. The observed structural patterns have implications beyond biomarker discovery. The presence of mucin-like flexible loops, amphipathic lipid-binding helices, immune-modulatory fragments, and redox-active peptides in diabetic saliva provides a molecular basis relevant to mucosa-mimetic biomaterials and biofabrication [22,23]. This study highlights peptide-centric motifs that emphasize biochemical properties essential for hydration, lubrication, immunological compatibility, and redox stability in engineered mucosal systems, contrasting with the current emphasis on mechanical properties in contemporary bioinks. Several limitations should be acknowledged. This study is a secondary reanalysis of an existing dataset, and the cohort size limits direct clinical generalization. Additionally, because all peptides result from controlled enzymatic digestion, no claims are made about endogenous peptide bioactivity or in vivo protease activity. Nevertheless, the consistency, coherence, and biological plausibility of the peptide-level findings strongly support their relevance for hypothesis generation and justify evaluation in larger, independent cohorts. This study did not conduct direct analytical comparisons between saliva- and blood-based peptide tests; therefore, no claims are made regarding their relative sensitivity or reproducibility. Blood is generally considered the clinical gold standard for T2D biomarker quantification because it has higher protein concentrations, established reference ranges, and consistent pre-analytical procedures. For saliva to become a viable alternative for metabolic monitoring, several developments are necessary. These include standardizing pre-collection protocols, developing normalization strategies that account for salivary flow and dilution, creating targeted peptide assays with defined limits of detection, determining the extent of inter-individual variability over time, and evaluating saliva in large longitudinal cohorts against established blood biomarkers such as HbA1c and fasting glucose. The current study advances this field by identifying consistent peptide-level signals in saliva that may be used for subsequent targeted validation and clinical certification.

## 4. Materials and Methods

### 4.1. Saliva Collection and Sample Preparation

All figures presented in this section were generated de novo from the peptide-centric DIA-NN reanalysis of the raw DIA-PASEF data and are distinct from the protein-centric and metaproteomic visualizations reported by Samodova et al. [11]. In the original ADDITION-PRO study, saliva samples were collected under standardized conditions to minimize confounding by food residues and oral debris. Participants were instructed to fast and to avoid eating, drinking (except water), smoking, or oral hygiene procedures prior to sample collection. Following collection, samples were centrifuged to remove intact cells and particulate material, thereby reducing the contribution of exfoliated epithelial cells and debris before proteomic processing. Accordingly, all downstream analyses and structural interpretations are confined to analytical and physicochemical inference, and no claims are made regarding endogenous peptide biology. This study is based on the reanalysis of the raw DIA-PASEF mass spectrometry data generated and published by Samodova et al. [11]. Each biological sample was processed once through digestion and LC–MS/MS analysis. No technical replicates of tryptic digestion or mass spectrometry acquisition were performed. DIA-PASEF acquisition combined with DIA-NN peptide-level quantification was used to ensure analytical consistency across samples, and downstream statistical analyses were conducted at the biological replicate level. In brief, 20 µL of saliva was mixed with 10 µL of lysis buffer containing 1% sodium deoxycholate (SDC), 100 mM Tris-HCl (pH 8.5), 10 mM TCEP, and 40 mM chloroacetamide. The mixture was heated at 99 °C for 10 min to denature the proteins and disrupt secondary structures, then subjected to 4 min of probe sonication to ensure complete homogenization. Proteins underwent a standard two-step digestion protocol. The samples were first incubated with Lys-C (1:250, 1 h, and 37 °C), followed by overnight digestion with trypsin (1:100 and 37 °C), as per the referenced protocol. Digestion was stopped by adding 10% TFA (1:10 and *v*/*v*), and peptides were clarified by centrifugation (17,000× *g*; 5 min). The supernatants were quantified by spectrophotometry and desalted using C18 StageTips. Approximately 200 ng of purified peptides per sample were loaded onto Evotips for LC–MS/MS analysis, following the preparation protocol described in the parent document. All peptides analyzed in this study were generated through controlled Lys-C/trypsin digestion and therefore represent digestion-derived analytical features rather than native, in vivo circulating peptides.

### 4.2. Liquid Chromatography–Mass Spectrometry (LC–MS/MS)

Peptide separation and analysis were performed using an Evosep One liquid chromatography system coupled with a Bruker timsTOF instrument (Billerica, MA, USA). The samples were analyzed using the 21-min “60 samples per day” protocol developed by Samodova et al. [11], enabling a high-throughput and comprehensive proteomic workflow. Chromatographic separation was achieved with an 8 cm PepSep C18 column (150 µm inner diameter; 1.5 µm particles), and ionization was facilitated by a CaptiveSpray source.

Data were acquired using DIA-PASEF with the following parameters:

- *m*/*z* acquisition range: 400–1200
- TIMS ion mobility range: 0.6–1.43 Vs/cm^2^
- Ramp/accumulation duration: 100 ms

DIA scheme: 32 × 25 Da windows, each acquired through 2 × 16-scan repetitions.

- Total cycle duration: approximately 1.8 s

This acquisition strategy combines ion mobility separation with wide-window DIA to achieve comprehensive peptide coverage within a short gradient.

### 4.3. Proteomic Data Processing (DIA-NN Reanalysis)

Raw DIA-PASEF files were reanalyzed with DIA-NN (v.1.8+) in library-free mode to improve peptide-level resolution. Database searches were performed using a hybrid FASTA that includes the reviewed (Swiss-Prot) proteome of Homo sapiens.

Peptide and Precursor Parameters: minimum peptide length: five amino acids; maximum peptide length: 40 amino acids; precursor mass range: 500–2000 Daltons; maximum allowable missed cleavages: two; enzymatic specificity: fully tryptic; fixed modification: carbamidomethylation of cysteine; and variable modifications: oxidation of methionine and acetylation at the N-terminus.

FDR and Normalization: FDR threshold: 1% at both the precursor and protein levels; interference correction: enabled; and RT-dependent normalization: enabled.

Initial quantification was performed at the precursor level using the neural network scoring model of DIA-NN. For peptide-centric analysis, the intensities of all confidently identified precursors corresponding to the same peptide sequence (including different charge states and missed cleavage variants) were subsequently aggregated to obtain peptide-level abundance estimates prior to statistical analysis.

### 4.4. Peptide-Centric Statistical Analysis (MetaboAnalyst 6.0)

All statistical analyses were performed at the peptide level, using quantitative outputs obtained directly from DIA-NN. In DIA-NN, precursor intensities for identical peptide sequences were aggregated for each peptide sequence to generate a single peptide-level abundance value, which served as the primary unit of analysis. No aggregation was performed at the protein level. Peptide abundance matrices were imported into MetaboAnalyst 6.0 for subsequent statistical analysis. Data were log_2_-transformed to stabilize variance and improve normality. Peptides identified in fewer than 70% of samples within each experimental group were removed to reduce sparsity. The remaining missing data were imputed using the k-nearest neighbors (KNN) method. Principal component analysis (PCA) was used for exploratory multivariate analysis to assess the overall sample structure and identify potential outliers. Differential abundance patterns of peptides between groups were analyzed using unsupervised hierarchical clustering and heatmap visualization, based on normalized peptide intensities. Clustering was performed using appropriate distance metrics and linkage methods to identify coherent peptide expression patterns associated with type 2 diabetes. Peptide clusters with differential abundance were annotated using UniProt and analyzed in the context of relevant biological pathways and physiological processes.

### 4.5. AlphaFold Structural Modeling of Full-Length Proteins and Mapping of Differentially Abundant Peptides

To determine whether peptides with differential abundance in T2D saliva displayed structural features relevant to bioink behavior, such as amphipathic helices, flexible regions, or accessible hydrophobic surfaces, a representative set of peptides was modeled using AlphaFold/ColabFold. Peptides were selected based on their biological significance, effect size, and potential influence on lubrication, matrix interaction, redox balance, or immunomodulation of bioprinted constructs. Structural predictions were generated using standard AlphaFold/ColabFold settings, yielding three-dimensional conformations with per-residue pLDDT confidence scores. Models were evaluated for secondary structure elements, flexibility, charge distribution, and solvent-exposed motifs relevant to bioprinting applications. The final models were presented in both ribbon and surface representations for comparative analysis.

## 5. Conclusions

In conclusion, this peptide-centric reanalysis demonstrates that type 2 diabetes reshapes the salivary environment through coordinated, structurally coherent peptide-level changes rather than wholesale proteome disruption. By integrating high-resolution DIA-NN peptide quantification with structure-informed physicochemical interpretation, this work advances salivary proteomics beyond descriptive protein lists toward a mechanistic understanding of metabolic disease at mucosal surfaces, opening new avenues for non-invasive diagnostics, longitudinal monitoring, and disease-informed biomaterial design.

## Data Availability

This study is a secondary analysis of publicly available mass spectrometry data originally generated by Samodova et al. [11]. The raw DIA-PASEF files are available via the ProteomeXchange Consortium (PRIDE) under accession number PXD051453.

## Supplementary Materials

The following supporting information can be downloaded at: https://www.mdpi.com/article/10.3390/ijms27042040/s1.
